# Unpaywall

**DOI:** 10.5195/jmla.2019.650

**Published:** 2019-04-01

**Authors:** Kerry Dhakal

**Affiliations:** Assistant Professor and Research and Education Librarian, Health Sciences Library, Ohio State University, Columbus, OH, dhakal.9@osu.edu

## INTRODUCTION

Finding research data and studies that are easily accessible for the purposes of furthering the sciences is the crux of much of what researchers do when they search the Internet and subscription databases. However, many studies and their data are still inaccessible without an institutional or individual subscription to a journal or subset of journals. In addition, many scholarly works are now being deposited into institutional repositories that are not necessarily known or aggregated into search engines consistently.

Consequently, finding these data and studies still presents a challenge. Many researchers will openly contact their colleagues at other institutions asking them to share access or send an article before even considering contacting their libraries to obtain it via the interlibrary loan (ILL) process, while others will use web tools such as ResearchGate, Academia.edu, or Twitter [1, 2]. The desire for immediate access to full-text articles is prevalent in today’s scientific community.

There are some tools that have aggregated data and studies from institutional repositories or open access (OA) websites and journals, such as OAIster, OpenDOAR, Google, and Google Scholar. All of these tools, however, include more than one step to access articles and are not comprehensive in their offerings. For example, some tools and search engines do a great job making articles legally and freely accessible from only certain disciplines [3].

Unpaywall, created by Jason Priem and Heather Piwowar of Impactstory in 2017, is a plug-in tool for individual users to access and find OA articles, regardless of where they are searching for those articles. It can be used in Chrome or Firefox browsers and helps the user identify articles that are freely available, regardless if the user is locating articles via institutional subscriptions or on the Internet.

Unpaywall is able to do this because it has created a data set of OA articles based on the 95 million articles that are included in the CrossRef website’s database of digital object identifiers (DOIs) and of freely available articles from 50,000 institutional repositories and databases [4, 5]. CrossRef is a not-for-profit organization that registers official DOIs for online journal articles [6]. Currently, Unpaywall has made over 20 million articles accessible to users through its tool. Unpaywall also has tools that libraries and institutions can integrate into their databases and institutional repositories, as well as build on using the oaDOI database [7].

## ACCESS TO ARTICLES

Unpaywall has different levels of usability depending on interests and the expected use of information. Individual users can submit a request using the Simple Query Tool on the website to determine if there is an OA version of an article or a list of articles that they are interested in [8]. A list of the DOIs for articles of interest and an email address can be submitted using this tool. Unpaywall will compare the DOIs in the list request and report back to the user. Users can also submit a list of up to 100,000 DOIs per day using the REST API tool, and the report will be sent to the email address provided [8]. Unpaywall recommends that users who want to request a report of more than 100,000 DOIs per day utilize the Database Snapshot feature. The purpose of using the Simple Query Tool, the REST API, and the Database Snapshot is to identify potential OA, full-text articles for a list of DOIs that have been submitted to Unpaywall.

Another tool in the Unpaywall toolbox is the plug-in. The plug-in identifies full-text articles when a user is searching the literature in a research database. Once the plug-in is loaded, a green or gray tab with a locked/unlocked icon appears to the right side of the screen to report if there is an OA version of the article available. A green tab identifies an article that does have an OA full-text article available for use, and a gray tab identifies an article that does not have an OA full-text article available. Clicking on the green tab will take the user to the full-text article. Once the plug-in is downloaded, there are options to change the colors of the tabs to match different types of OA content, including content from institutional repositories and prepublication websites.

Unpaywall in now integrated into three major databases: Dimensions, Scopus, and Web of Science. This helps searchers more easily identify OA content in those databases using Unpaywall’s Data Feed tool that vendors subscribe to [9]. Data Feed requires registration and a subscription fee, which is based on the size of the organization and intended use of the data. One reason that vendors would subscribe to the Data Feed tool, rather than use the Database Snapshot, is that the Data Feed tool is updated daily. The Database Snapshot tool is only updated a few times a year [10]. There are a few different ways that libraries can integrate Unpaywall into their link resolvers, including SFX, 360 Link, and Primo and by creating a unique link [11].

Unpaywall is expanding its reach even further by developing a tool for the non-scientist community that will translate scientific journal articles into plain language information [9].

## AUDIENCE

Anyone who is interested in accessing research articles from repositories, databases, publishers, or others should consider using Unpaywall. Most scientific researchers who regularly search for articles on the Internet will find this resource to be very helpful. Librarians will also find that the tool is user-friendly through uploading to institutional link resolvers that can be used in database searching. For example, it will provide additional information to library users about which articles in the library’s subscriptions are OA by presenting a green, unlocked lock icon tab to the right of the screen so that users know this article is openly available to them. Library users can then either click on the Unpaywall green unlocked icon to access the full-text of the article or access the article more traditionally by using the links that the library provides.

Users can actually gain additional access to content because of the ease of using the Unpaywall color-coded tabs. Researchers and practitioners who are not affiliated with any institutional libraries will find this tool very accommodating as well. For all users, it helps take the guesswork out of accessing OA articles, since Unpaywall only accesses OA content, unlike articles that can be found on websites like ResearchGate or Academia.edu, where scholars may upload articles that are not necessarily legally OA.

## USABILITY AND TECHNICAL ASPECTS

Unpaywall is very easy to use because the identifier of OA versus non-OA articles automatically pops up on the search screen when an article is being reviewed. One reviewer found that the tabs do not always appear, due to preset pop-up blockers and privacy settings [7]. Administrative settings may need to be checked if someone is using the plug-in on computers in hospitals, especially if those computers are using Internet Explorer as a browser.

## COMPARISON TO OTHER RESOURCES

There are other tools on the Internet to find OA articles, including the OA Button and Kopernio. All of these tools have a plug-in feature that can be added to a web browser. There are several differences between the Unpaywall plug-in and the OA Button plug-in. The Unpaywall plug-in instantly appears when an article has been found and identifies the article by color immediately, so that the user knows if it is OA or not, while the OA Button plug-in does not have this feature. Instead, when the searcher finds an article, the OA Button is an icon at the top right on the web browser that needs to be used to be taken to the full-text article, if an OA version exists.

Another helpful feature of the Unpaywall plug-in is that it allows users to change the colors of the plug-in tab so that users can know what level of OA the articles are (i.e., gold, green, or white). This feature of changing colors is not available with OA Button. OA Button does provide an option to email the author of an article that is not OA asking them to consider sharing the article and/or making the article available to users via an OA venue.

## COST

The Simple Query Tool, REST API, Database Snapshot, and the plug-in tool are all available without charge for use and download. As previously noted, the Data Feed tool is fee-based because it is updated daily, and it costs more to harvest the data on such a frequent basis [10].

## CONCLUSION

Unpaywall is one of the newer plug-in extensions that have been created to help access research data and studies by identifying articles as freely and legally OA, without requiring a change in search technique. They have been integrated into subscription databases and offer link resolver tools for libraries so they can join their mission of ensuring access to research articles for all.
